# Evaluating the Effectiveness of a Front Windshield Sticker Reminder in Reducing Texting while Driving in Young Adults

**DOI:** 10.7759/cureus.691

**Published:** 2016-07-14

**Authors:** Austin Rohl, Sven Eriksson, David Metcalf

**Affiliations:** 1 Medicine, UCF College of Medicine; 2 Institute for Simulation & Training, University of Central Florida

**Keywords:** texting while driving, distracted driving, mindfulness

## Abstract

Texting while driving is a dangerous activity that is on the rise in the United States (U.S.). Since 2011 there has been a 17% increase in the number of people injured in a motor vehicle crash involving a distracted driver. Bans on the act of texting and driving have already taken place in 46 states in the U.S., but studies have shown that they are ineffective. An unstudied method of reducing texting while driving is sticker reminders. Sticker reminders have already been proven to be an effective intervention in the realm of driver safety; one study found that a “Buckle-Up” dashboard sticker doubled the use of safety belts by front seat passengers. In this study, 104 medical students aged 21 - 29, from the University of Central Florida (UCF) College of Medicine were randomly divided into two groups, an interventional group receiving a “Drive in the Moment” windshield sticker, and a control group not receiving the sticker. Both groups took a pre- and post-survey that recorded self-reported texting and driving frequency. The results showed that the sticker intervention significantly reduced reported rates of sending texts while driving, reading texts while driving, and using social media while driving (p < 0.05). The results of this study suggest that a novel sticker-intervention could potentially serve as a point of attack when addressing the growing and dangerous texting while driving the epidemic.

## Introduction

Distracted driving is a major public health concern, responsible for 3,179 deaths and 431,000 injuries in the United States in 2014 alone [1]. The leading distractor involved in these motor vehicle accidents is cell phones, and particularly the practice of sending and receiving text messages while behind the wheel [1]. Studies comparing the reaction times of drivers using cell phones to drivers under the influence of alcohol have shown that using a cell phone is equivalent to a blood alcohol content of 0.8, which is legally drunk in many states [2]. The normal text involves lending your eyes to your phone for approximately 5 seconds, which is enough time to drive 360 feet, the length of an entire football field [3]. The percentage of drivers text-messaging or visibly operating a handheld device increased from 1.7% in 2013 to 2.2% in 2014 [4]. Of note, since 2007 young drivers have been observed using handheld devices at higher rates than older drivers [4]. Unfortunately, ten percent of all drivers 15 to 19 years old involved in fatal crashes had been reported as distracted at the time of the incident [4]. Another frightening statistic is that nationally there are approximately 660,000 Americans using their cell phones while driving at any given time [4].

It is clear that cell phone use while driving is a dangerous activity that is on the rise in the U.S. There have been several attempts to reduce the impact of cell phone use on driving. Bans on the act of using cell phones while driving have already taken place in 46 states, but studies suggest that these have had a negligible effect on reducing crashes linked to texting while driving [5]. One study showed that negative and fear-based public service announcements actually, led to an increase in likeliness to engage in texting while driving behaviors on a follow-up survey [6]. One possible means of reducing the texting while driving rates is to use the cell phone technology itself, either by implementing hands-free texting technology or apps that prevent texting while in a car. These advancements in preventative technology, however, have seen mixed results. One study found that hands-free texting via Apple’s Siri voice to text service was no less distracting and negatively impacted reaction times just as much as hands on texting [7]. A major concern for these types of innovations in the cell phone technology is that not everyone has access to these specific technologies and apps. Reports have shown that only 68% of mobile users are on a smartphone platform such as Apple and Android, leaving 32% of users without access to these apps that attempt to reduce texting while driving [8]. The price of the smartphone aside, the price of some of the app solutions themselves are barriers for those who can’t afford them, with some of the most notable apps such as *Cellcontrol* charging over $100 to initiate their service [9]. Even though these apps are available, there is no way to regulate adherence to the usage of an app that disables texting while in a car. Given these cost and access barriers, there is a need for a simpler solution, perhaps not involving the use of cell phone technology.

One simple method of reducing texting while driving that has been suggested, but has yet to be evaluated is sticker reminders [10]. Sticker reminders are a proven simple, yet effective method of invoking behavioral change, as was demonstrated in a “Buckle-Up” dashboard sticker study, which doubled the use of safety belt use by front seat passengers [11]. Sticker reminders have also been an effective intervention in the hospital setting, being used to improve things like child abuse detection, smoking cessation, catheter-associated urinary tract infections, venous thromboembolism prophylaxis, and antibiotic prescribing, among others [12-15]. One mechanism behind the efficacy of such sticker campaigns is that they evoke an increased state of mindfulness [16]. Mindfulness is the process of bringing one’s attention and awareness to the present moment [17]. Given the effectiveness of sticker interventions that have been applied to a wide variety of preventative health measures a similar intervention may be useful is addressing the epidemic of texting while driving.

The purpose of this study was to assess the efficacy of a simple sticker reminder intervention in reducing rates of texting while driving in young drivers. The goal of the intervention was to provide a visual reminder to pay attention to your surroundings and to avoid multi-tasking while driving. We assessed how this intervention affected the behaviors of texting while driving, and through what mechanism this intervention brought upon the behavioral change. More specifically, we were attempting to evoke an increased state of mindfulness with the idea that the drivers, would be more engaged in the present moment, less likely to use their phones to communicate with others, and more aware of the risks involved with texting while driving. The ultimate goal of this study was to develop a simple and effective intervention that would empower physicians, lawmakers, and car manufacturers to prevent the thousands of deaths and hundreds of thousands of injuries caused by texting while driving each year.

## Materials and methods

### Study design and participants

The study consisted of a pre-survey, intervention, and an identical post-survey design. There were 103 medical students (ages 21 - 29) from the University of Central Florida College of Medicine who participated in this study. Participants were included in the study if they held a valid driver's license and had access to a vehicle. The pre-survey was administered in person. Following the pre-survey, participants were randomly assigned to either a sticker group or a control group. The sticker group received a windshield sticker and applied it to their vehicles. The control group did not receive a sticker. Three weeks later, after resuming normal driving behaviors, both groups were contacted and completed the post-survey online. Participants received monetary compensation for participation in the pre-survey and again in the post-survey. All participants were given informed consent, and the study was approved by the University of Central Florida Institutional Review Board (IRB).

### Intervention

**Figure 1:**
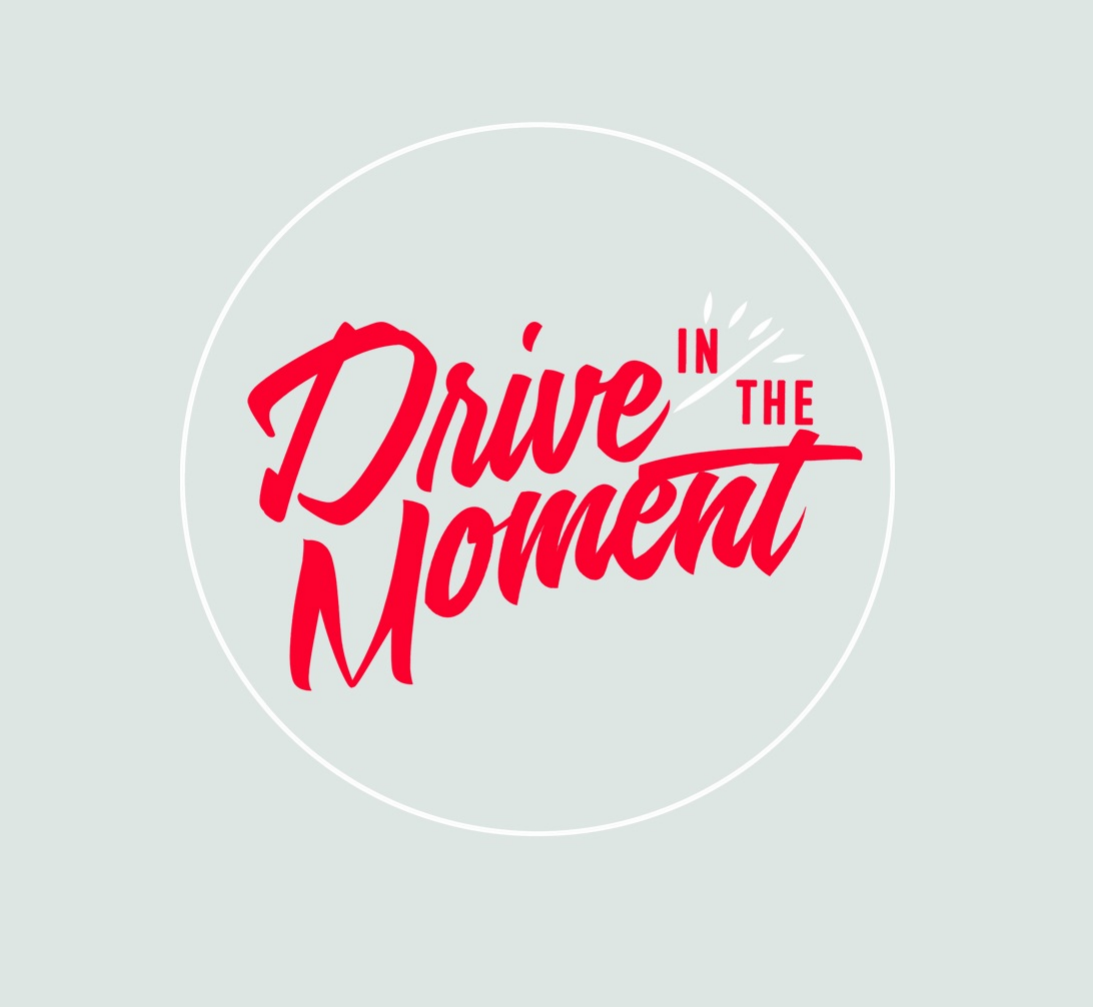
Sticker Design 2.5cm x 2.5cm with clear background

**Figure 2:**
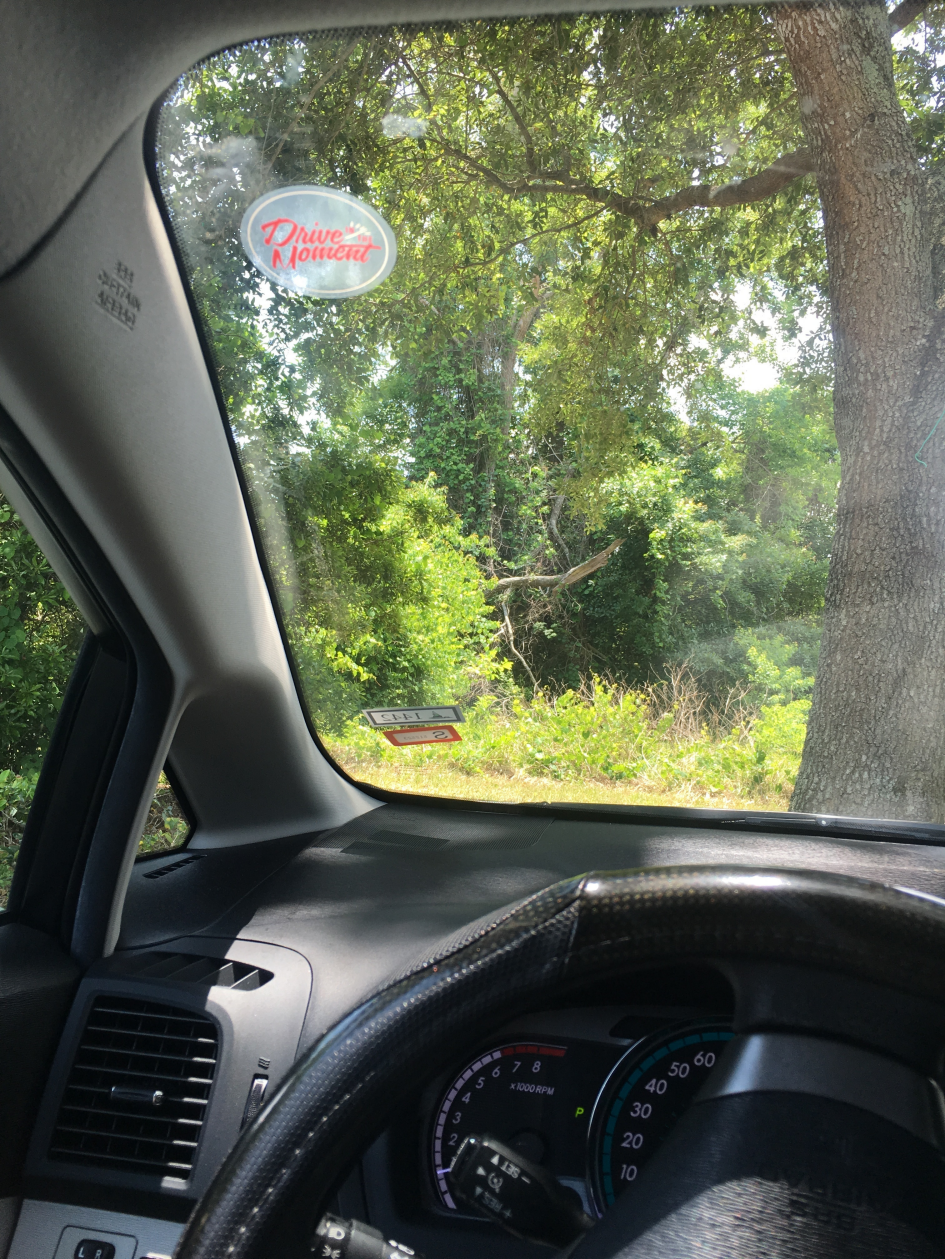
Example of the sticker inside a vehicle

Immediately following the completion of the pre-survey, a proctor accompanied sticker group participants to their vehicles and applied a 3” x 2.5” adhesive sticker, which displayed the words “Drive in the Moment” to the inside of the driver side windshield in the upper left quadrant. This way the driver could see the sticker, without obstructing their field of vision (Figure 1 and Figure 2). Upon receiving the sticker, the participants were also verbally reminded that it should serve as a reminder to not text and drive. The sticker group participants then resumed using their vehicles for three weeks, after which they were emailed a link to complete the post-survey. The only change in the protocol for the control group was that they did not receive a windshield sticker and were not accompanied to their vehicles for which to apply a sticker. All other procedures including compensation, study timing, and survey administration were identical for both groups.

### Pre- and post-surveys

The pre and post surveys contained demographic measures of age, sex, ownership of driver’s license, ownership of cell phone, mobile model, primary access to a vehicle, and driving frequency. The behavior of texting while driving was assessed in two self-reported retrospective modalities: sending texts and reading texts. This two-dimensional model of texting has been shown to be an effective captor of texting while driving habits, as some people are more likely to read texts than send them, and vice-versa [18, 19]. Participants were asked, “On average, in the past three weeks, how many texts did you send (read) while driving?,” with response options on a seven-point scale (1 = more than 10 a day, 2 = 5 - 10 a day, 3 = 2 - 4 a day, 4 = at least one a day, 5 = 3 - 6 a week, 6 = 1 - 2 a week, 7 = less than 1 - 2 a week). This question was adapted from previously verified surveys that have been used in comparable studies monitoring texting while driving behaviors [18, 20]. Ordinal frequency measures of texting while driving were used in this study and had been shown to be a more accurate method of gathering self-reported cell phone use data than a continuous frequency measure [21]. The questionnaire also included a question about social media use while driving. This item read “On average, in the past three weeks, how many times did you open and view social media apps (i.e. Facebook, Instagram, Snapchat, etc.) while driving?,” and used the same 7-point scale as the texting items. The surveys also assessed mindfulness by incorporating the Cognitive and Affective Mindfulness Scale-Revised (CAMS-R) mindfulness questionnaire, a previously validated assessment of overall mindfulness [22]. The CAMS-R questionnaire consists of 12 items assessing individual differences in capacity to focus attention on present moment experience, as well as awareness and acceptance of thoughts and feelings [22]. The full survey is provided in the appendix. Independent samples t-tests were performed to analyze pre-survey ordinal data from the texting section of the surveys. Paired samples t-tests were performed to assess compare pre- and post-survey data. Pearson correlation coefficients were calculated to assess the relationship of mindfulness to texting while driving frequency. All statistical analyses were performed using IBM SPSS software.

## Results

Table 1 shows the pre-survey scores of the control and sticker groups. An independent samples t-test showed that there was no significant difference in initial pre-survey scores between the control group and sticker group among all three modalities: sending, reading, and using social media while driving.

**Table 1:** Pre-survey Group Analysis T-test for Equality of Means *^1^* *Scores reported in a 1-7 scale Likert Scale, higher numbers indicate less sending/reading/using social media while driving.*

Variable	Group	Pre-survey Mean Score^1^	Pre-survey Mean Score^1^ Difference (Sticker - Control)	P Value Mean Score Difference
Sending Texts	Control (N=49)	4.61 *(2.110)*	0.036	0.930
	Sticker (N=54)	4.65 *(2.030)*		
Reading Texts	Control (N=49)	3.88 *(1.844)*	-0.044	0.902
	Sticker (N=54)	3.83 *(1.778)*		
Using Social Media	Control (N=49)	5.18 *(2.157)*	0.131	0.763
	Sticker (N=54)	5.31 *(2.239)*		

Table 2 shows the mean differences of pre-survey versus post-survey scores in both the control and sticker group. Paired samples t-tests showed that there were significant decreases in all three modalities (sending texts, reading texts, and using social media while driving) among the sticker group (p < 0.05).* *Analysis of the control group showed no statistically significant differences in pre-survey and post-survey scores.

**Table 2:** Paired Samples T-Test ^1^Scores reported in a 1-7 scale Likert Scale, higher numbers indicate less sending/reading/using social media ^2^A positive mean score difference indicates the participant reported less sending/reading/using social media after the sticker intervention. Higher numbers indicate a stronger reduction in these behaviors after the sticker intervention. *Significant at α= 0.05 level

Variable	Group	Pre-survey Mean Score^1^	Post-survey Mean Score^1^	Mean Score Difference^2^ (Post – Pre)	P Value Mean Score Difference
Sending Texts	Control (N=49)	4.61 *(2.110)*	4.82 *(1.845)*	0.204 *(1.118)*	0.207
	Sticker (N=54)	4.65 *(2.030)*	5.07 *(2.100)*	0.426 *(1.449)*	0.035*
Reading Texts	Control (N=49)	3.88 *(1.844)*	4.20 *(1.732)*	0.327 *(1.214)*	0.066
	Sticker (N=54)	3.83 *(1.778)*	4.39 *(1.795)*	0.556 *(1.369)*	0.004*
Using Social Media	Control (N=49)	5.18 *(2.157)*	5.27 *(1.868)*	0.082 *(1.441)*	0.693
	Sticker (N=54)	5.31 *(2.239)*	5.70 *(2.160)*	0.389 *(1.393)*	0.045*

Table 3 and Table 4 show Pearson Correlation coefficients between mindfulness score and the three texting while driving modalities. Because there was no significant difference in pre-survey and post-survey mindfulness scores, pre-survey mindfulness scores were used empirically. Mindfulness scores showed no significant correlation with any of the measured modalities of texting while driving. In both the control group and sticker group, all three measured modalities of texting while driving, including reading texts, sending texts, and using social media while driving were all significantly correlated with each other (p < 0.01).

**Table 3:** Pearson Correlation Tests for Pre-survey Scores and Mindfulness in the Control Group **Correlation is significant at the 0.01 level (2-tailed)

		Mindfulness Score (pre-survey)	Sending Texts (pre-survey)	Reading Texts (pre-survey)	Using Social Media (pre-survey)
Mindfulness score (pre-survey)	Pearson Correlation	1	.083	.218	.012
	Sig. (2-tailed)		.574	.137	.934
	N	48	48	48	48
Sending Texts (pre-survey)	Pearson Correlation	.083	1	.834^**^	.570^**^
	Sig. (2-tailed)	.574		.000	.000
	N	48	49	49	49
Reading Texts (pre-survey)	Pearson Correlation	.218	.834^**^	1	.582^**^
	Sig. (2-tailed)	.137	.000		.000
	N	48	49	49	49
Using Social Media (pre-survey)	Pearson Correlation	.012	.570^**^	.582^**^	1
	Sig. (2-tailed)	.934	.000	.000	
	N	48	49	49	49

**Table 4:** Pearson Correlation tests for Pre-survey Scores and Mindfulness in the Sticker Group **Correlation is significant at the 0.01 level (2-tailed)

		Mindfulness Score (pre-survey)	Sending Texts (pre-survey)	Reading Texts (pre-survey)	Using Social Media (pre-survey)
Mindfulness score (pre-survey)	Pearson Correlation	1	-.058	.058	.023
	Sig. (2-tailed)		.681	.683	.870
	N	52	52	52	52
Sending Texts (pre-survey)	Pearson Correlation	-.058	1	.825^**^	.619^**^
	Sig. (2-tailed)	.681		.000	.000
	N	52	54	54	54
Reading Texts (pre-survey)	Pearson Correlation	.058	.825^**^	1	.639^**^
	Sig. (2-tailed)	.683	.000		.000
	N	52	54	54	54
Using Social Media (pre-survey)	Pearson Correlation	.023	.619^**^	.639^**^	1
	Sig. (2-tailed)	.870	.000	.000	
	N	52	54	54	54

## Discussion

This study assessed the efficacy of a simple sticker intervention in reducing rates of self-reported texting while driving. Initially, participants in both the control and sticker groups proved to have no significant difference in their baseline texting while driving habits, indicating that the control and sticker groups were not different in texting while driving habits before the intervention. The mean score difference from pre-survey to post-survey showed that the windshield sticker intervention yielded significant reductions in self-reported texting while driving behaviors. In the sticker group, the mean score difference for sending texts was 0.426 (p = 0.036), which means that they sent fewer texts while driving with the sticker in place. Similar significant improvements occurred in the sticker group for the reading texts and using social media while driving modalities. While the control group also showed mean difference improvements in all three modalities, none of these improvements were statistically significant. The sum of these findings indicates that the windshield sticker reminder used in this study could have possibly lead to the reduced texting while driving behaviors.

When the sticker used in this study was designed, with the words “Drive in the Moment” printed on it, the aim was to increase the driver’s mindfulness while in the vehicle. Mindfulness expert Jon Kabat-Zinn proposed in 2005 that people’s reliance on mobile technology could be lowered by increasing mindfulness [23]. Feldman et al. suggests through his study that drivers lower in mindfulness would be more likely to report more frequent texting while driving, and also implicated that this relationship between mindfulness and texting while driving could be mediated by emotion and attention regulation motives [16]. While Feldman's* *showed through path analysis that mindfulness is a predictor of texting while driving behaviors, our study found no such significant relationship. This contrasting finding in our study could be due to lack of incorporation of important co-variants in analyses such as emotion regulation motives and contextual variables that played an important independent role in predicting texting while driving behaviors in Feldman et al.* *[16]. It is possible that while the sticker intervention was effective at reducing texting while driving behaviors, it may not have done so by evoking mindfulness. Many studies that have used a mindfulness-based intervention have instead utilized guided lessons, MBSR (mindfulness based stress reduction) techniques, and breath awareness techniques to induce increased levels of mindfulness [24-26]. A possible avenue for further research could involve texting while driving behaviors before and after a traditional mindfulness training program, and comparing it to this study.

This study also demonstrated a significant correlation between the use of social media while driving and rates of texting while driving. This correlation indicates that apps and programs to eliminate texting while driving, may not specifically be an effective means of reducing injury and death, as those who text while driving are just as likely to distract themselves with social media instead. Social media use should, therefore, be examined in all future texting while driving studies. Cook 2011has already shown that mobile internet access while driving is related to traffic citations and accidents, and is just as important of a factor as texting while driving [27]. Given the rise in data availability and prevalence of smartphones, it is important that social media use while driving is considered as a variable with the same dangerous impact as texting while driving.

### Study limitations

A large limitation to this study lies in the challenge to properly interpret the effect of the results in real practice. While there was a significant reduction in texting while driving, it is represented as a fraction of the texting while driving frequencies on the 1-7 scale. It is, therefore, a challenge to accurately interpret exactly how many or how less frequently texts were being sent and read, and if this amount is enough to cause an effect that reduces a driver’s risk of collision. Not being able to identify how many accidents the study could prevent will always be a limitation to the interpretation of the true impact of the results. The study population of medical students may be limiting to the generalizability of the study results to all young adults. The usage of self-reported texting while driving behaviors are another limitation. Because texting while driving is considered a reckless act there may have been under-reporting both before and after the intervention.

Another limitation of the study is the length of the intervention. While even three weeks of reduced texting while driving could be potentially life-saving, it is possible that the reduction in texting while driving behaviors could have been due to the mere novelty of participation in a texting while driving study, and that the period of lowered reduced behaviors might have ended had the study continued. In the 1987 study of the effectiveness of the “Buckle-Up” dashboard sticker, Thayer found that seatbelt use dropped significantly after the stickers were withdrawn for a two-week period, but when the stickers were reinstated, belt usage went back to previous high levels [6]. In future studies, a period of sticker withdrawal and further recording of texting while driving behaviors should be collected and analyzed. Future directions of this study should focus on the long-term effects of a sticker intervention, to evaluate if the sticker reminder holds its effectiveness over time or if it is a transient effect created by the novelty of the initial sticker placement. Further studies could also look into the effectiveness of the popular AT&T “No Text on Board” sticker campaign, in which participants place a bumper sticker on their car indicating they have pledged not to text and drive. Pledge programs have already proven effective in efforts such as teenage sexual abstinence and public goods distribution, and a detailed look into the effectiveness of several existing no texting pledges including “No Text on Board” could be evaluated alongside the usage of a sticker indicating the completed pledge [28-29]. It remains to be seen what the effect of a larger anti-texting while driving sticker intervention would have on accident rates in an area of study. 

## Conclusions

With rates of texting and driving on a steady increase, the exploration of novel solutions, like this sticker intervention, is necessary to prevent deadly car accidents that claim the lives of thousands every year. A simple sticker intervention in this study was effective at reducing self-reported texting while driving behavior, at least temporarily. Mindfulness scores did not change before and after the sticker intervention, and we found no correlation between texting while driving and mindfulness at baseline. Future studies are warranted to investigate the longevity of the observed sticker effect, as well as the real life implications of this reported reduction in behaviors. 

Just like the warning light for seat-beltless drivers in newly made vehicles, sticker reminders could be built into the windshields of cars, serving as a constant visual reminder not to text while driving. Insurance companies could perhaps give discounts for drivers who have anti-texting stickers if they are found to reduce this dangerous behavior. The texting while driving epidemic needs to have a point of attack; this sticker intervention could serve as a simple yet effective first move.

## Figures and Tables

**Figure 3:**
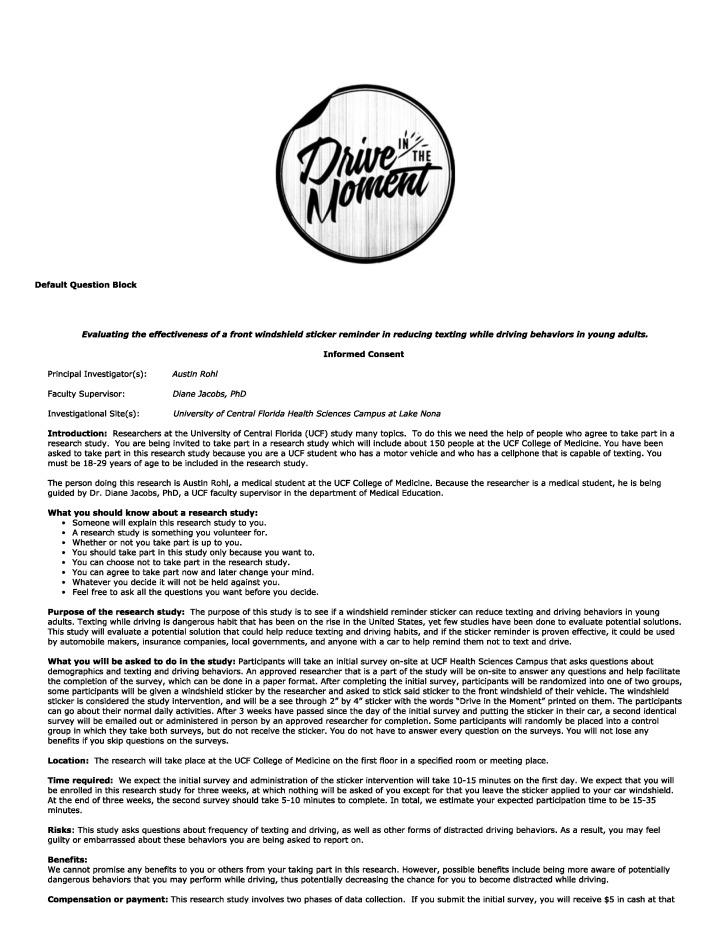
Survey page 1

**Figure 4:**
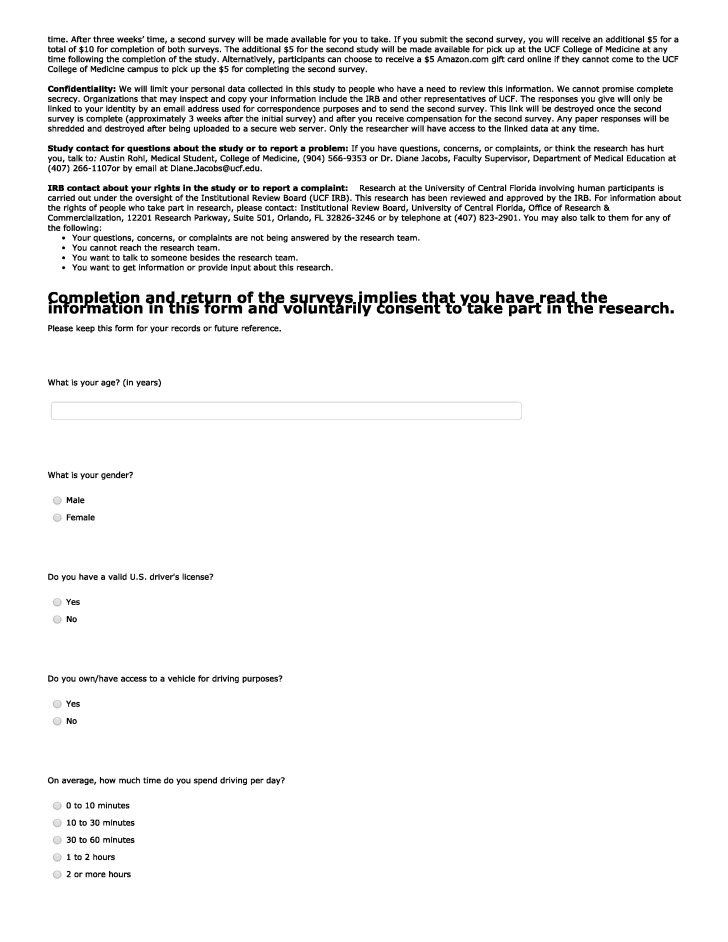
Survey page 2

**Figure 5:**
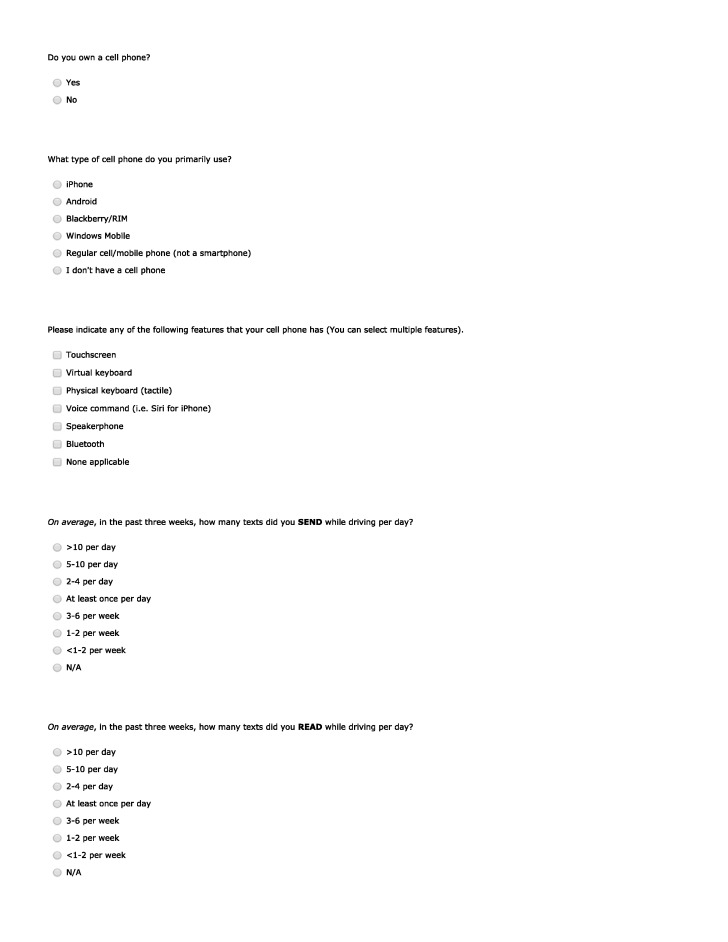
Survey page 3

**Figure 6:**
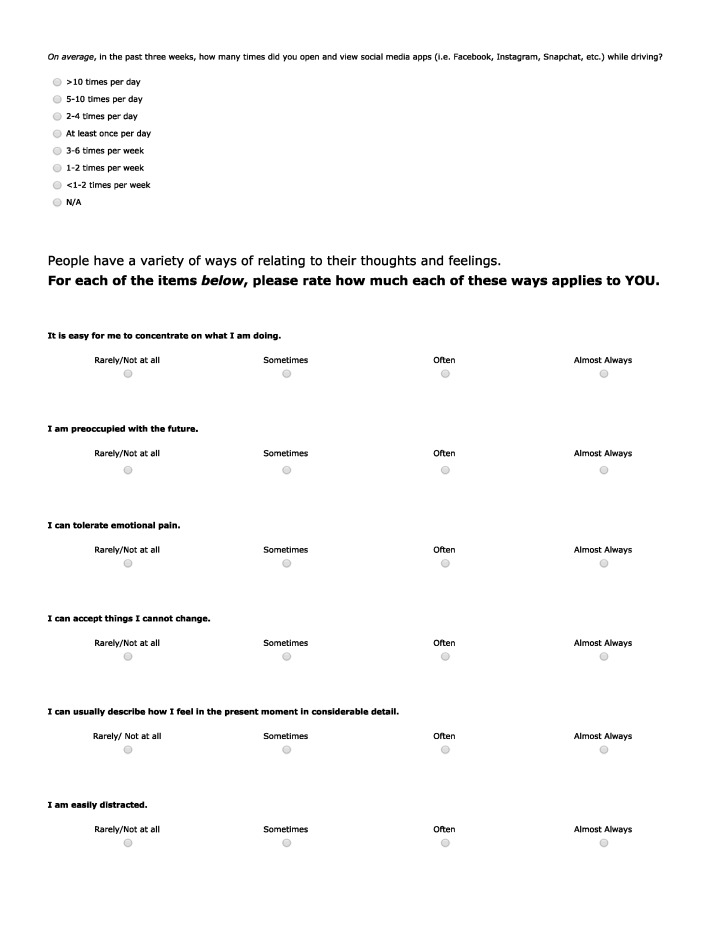
Survey page 4

**Figure 7:**
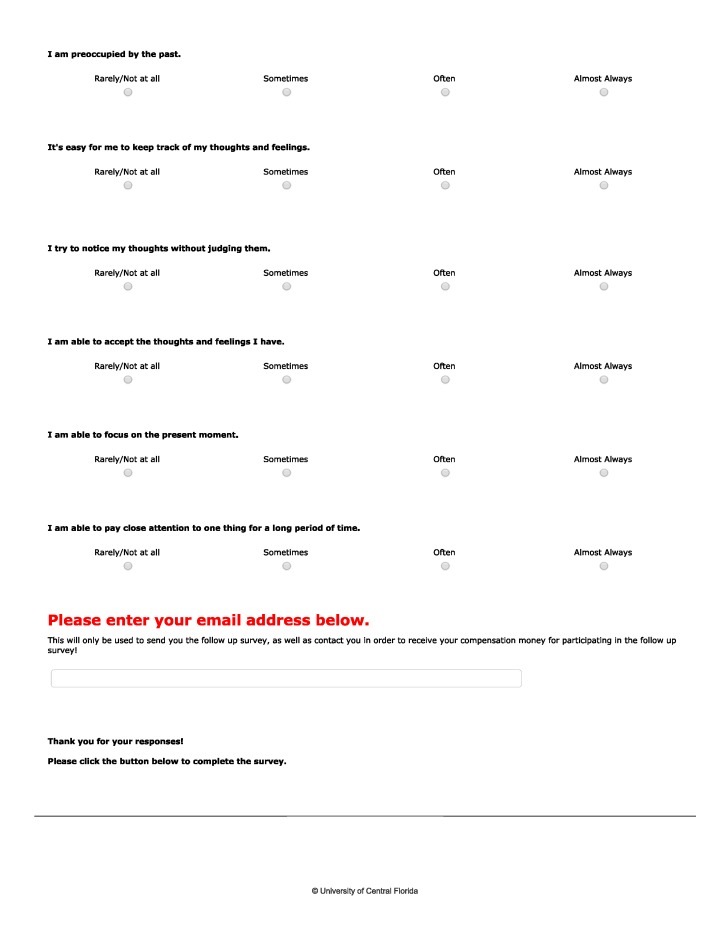
Survey page 5

## Appendices

Survey used in study:
